# SlARF10, an auxin response factor, is involved in chlorophyll and sugar accumulation during tomato fruit development

**DOI:** 10.1093/jxb/ery328

**Published:** 2018-09-13

**Authors:** Yujin Yuan, Lihua Mei, Mengbo Wu, Wei Wei, Wei Shan, Zehao Gong, Qian Zhang, Fengqing Yang, Fang Yan, Qiang Zhang, Yingqing Luo, Xin Xu, Wenfa Zhang, Mingjun Miao, Wangjin Lu, Zhengguo Li, Wei Deng

**Affiliations:** 1 School of Life Science, Chongqing University, Chongqing, China; 2 State Key Laboratory for Conservation and Utilization of Subtropical Agro-bioresources/Guangdong Provincial Key Laboratory of Postharvest Science of Fruits and Vegetables, College of Horticulture, South China Agricultural University, Guangzhou, China; 3 School of Chemistry and Chemical Engineering, Chongqing University, Chongqing, China; 4 Horticulture Research Institute, Sichuan Academy of Agricultural Sciences, Chengdu, China

**Keywords:** ARF10, auxin, chlorophyll, fruit, starch, sugar, tomato

## Abstract

The photosynthesis of green tomatoes contributes to fruit growth and carbon economy. The tomato auxin response factor 10 (SlARF10) belongs to the ARF family and is located in nucleus. In this study, we found that *SlARF10* was highly expressed in green fruit. Overexpression of *SlARF10* in fruit produced a dark-green phenotype whilst knock-down by RNAi produced a light-green phenotype. Autofluorescence and chlorophyll content analyses confirmed the phenotypes, which indicated that *SlARF10* plays an important role in chlorophyll accumulation. Overexpression of *SlARF10* positively affected photosynthesis in both leaves and fruit. Furthermore, *SlARF10*-overexpression lines displayed improved accumulation of starch, fructose, and sucrose in fruit, whilst *SlARF10*-RNAi lines showed decreased accumulation of starch and sucrose. Regulation of *SlARF10* expression altered the expression of *AGPase* starch biosynthesis genes. SlARF10 positively regulated the expression of *SlGLK1*, *POR*, *CBP1*, and *CBP2*, which are related to chlorophyll metabolism and regulation. Electrophoretic mobility shift assays confirmed that SlARF10 directly targets to the *SlGLK1* promoter. Our results thus indicate that *SlARF10* is involved in chlorophyll accumulation by transcriptional activation of *SlGLK1* expression in tomato fruit, and provide insights into the link between auxin signaling, chloroplast activity, and sugar metabolism during tomato fruit development.

## Teaser

*SlARF10* plays an important role in the chlorophyll accumulation and photosynthesis of tomato plants, and regulation of its expression affects the starch, fructose, and sucrose content of fruit.

## Introduction

Tomato (*Solanum lycopersicum*), a nutrient-rich multicarpellar berry with strong adaptability and high yield, has become the world’s second largest vegetable crop (Tanksley, 2004). It has also become the research model species for fleshy fruits, due to its short life cycle, self-pollination, and the ease with which mechanical crossing and genetic transformation can be conducted (Klee and Giovannoni, 2011).

Fruit development can be divided into three main stages (Ho and Hewitt, 1986). The first stage is characterized by intense mitotic activity, with an increase in cell number and starch accumulation (Ho, 1996). Cell enlargement associated with the degradation of starch into soluble sugars is characteristic of the second stage (Schaffer and Petreikov, 1997), whilst the third stage corresponds to fruit ripening, which is associated with the conversion of chloroplasts into chromoplasts, and the accumulation of carotenoids, sugars, organic acids, and volatile aromatic compounds in the cells (Klee and Giovannoni, 2011). The accumulation of soluble solids in ripening tomatoes is related to the starch level in immature and mature green fruit (Davies and Cocking, 1965). It has been reported that 10–15% of the total carbon and of the net sugar accumulation during fruit development comes from the photosynthetic activity in the fruit itself (Tanaka *et al.*, 1974; Obiadalla-Ali *et al.*, 2004). Hence, chloroplast development and the photosynthetic activity of green fruit affect the composition and quality of ripening tomatoes (Nadakuduti *et al.*, 2014).

Several genes influence the development of fruit chloroplasts and the subsequent quality of ripening fruit in tomatoes. The *DE-ETIOLATED 1*/*high pigment 2* (*DET1*/*hp2*) and *UV-DAMAGED DNA-BINDING PROTEIN 1*/*high pigment 1* (*DDB1*/*hp1*) genes encode negative regulators of photomorphogenesis. Down-regulation of *DET1/hp2* and *DDB1/hp1* increases the number of chloroplasts and plastid compartment size, thereby leading to higher levels of chlorophyll and carotenoids in the fruit (Liu *et al.*, 2004; Kolotilin *et al.*, 2007; Rohrmann *et al.*, 2011). GOLDEN2-LIKE (GLK) transcription factors are required for the regulation of chloroplast and chlorophyll levels (Waters *et al.*, 2008). Tomato contains two *GLK*s, namely *SlGLK1* and *SlGLK2*, which encode functionally similar peptides. Differential expression renders *SlGLK1* more important in leaves and *SlGLK2* more important in the fruit. The latitudinal gradient of *SlGLK2* expression across the axis of the fruit results in the typical uneven coloration of green and ripe wild-type tomatoes (Nguyen *et al.*, 2014). Tomato *ARABIDOPSIS PSEUDO RESPONSE REGULATOR 2-LIKE* (*SlAPRR2-like*) is the closest global relative of *SlGLK2*. Overexpression of the *APRR2-like* gene in tomatoes produces larger and more numerous chloroplasts, and consequently higher chlorophyll levels in green fruit and higher amounts of carotenoids in red ripening fruit (Pan *et al*., 2014). Two Class I KNOTTED1-LIKE HOMEOBOX (KNOX) proteins, namely TKN2 and TKN4, positively influence *SlGLK2* and *SlAPRR2-LIKE* expression to promote chloroplast development in tomato fruit (Nadakuduti *et al.*, 2014).

Phytohormones have been reported to be involved in chloroplast development and the quality of ripening fruit (Martineau *et al*., 1994; Galpaz *et al.*, 2008; Sagar *et al.*, 2013). Studies on the auxin signaling transduction pathway have indicated that auxin response factors (ARFs) are required for auxin-dependent transcriptional regulation in plants, and they can function as either transcriptional activators or repressors of auxin-responsive genes (Ren *et al.*, 2011). Most ARF proteins contain an N-terminal DNA-binding domain (B3), which is involved in transcription of auxin-response genes, a middle region acting as an activation domain or repression domain, and a C-terminal dimerization domain that requires the formation of heterodimers or homodimers (Zouine *et al.*, 2014). An increasing number of studies have demonstrated that *ARF*s play important roles in many developmental processes in tomato (Krogan *et al.*, 2012; Wang *et al.*, 2012; Guan *et al.*, 2013; Ckurshumova *et al.*, 2014; Liu *et al.*, 2014*a*, 2014*b*; Zhang *et al.*, 2015). *SlARF7* acts as a negative regulator of fruit set and development in tomato (De Jong *et al.*, 2009). *ARF6* and *ARF8* have important roles in controlling flower growth and development (Liu *et al.*, 2014*a*). *SlARF9* is required for the regulation of cell division during early tomato fruit development (De Jong *et al.*, 2015), *SlARF3* is involved in the formation of epidermal cells and trichomes (Zhang *et al.*, 2015), and *SlARF4* controls the accumulation of chlorophyll and starch in the fruit (Jones *et al.*, 2002; Sagar *et al.*, 2013). The influence of *SlARF4* on fruit chlorophyll accumulation seems to be mediated through the transcriptional up-regulation of *SlGLK1* in the fruit (Sagar *et al.*, 2013).

Hendelman *et al.* (2012) reported that *SlARF10* is post-transcriptionally regulated by Sl-miR160, and constitutive expression of *mSlARF10* (Sl-miR160a-resistant version) produced narrow leaflet blades, sepals, and petals, and abnormally shaped fruit in tomato. Repression of *SlARF10* expression by Sl-miR160 is essential for auxin-mediated blade outgrowth and early fruit development (Hendelman *et al.*, 2012). In the present study, the functions of *SlARF10* were studied in the development of tomato fruit, and it was found to be involved in chlorophyll and sugar accumulation. This study expands our understanding of the functions of ARFs during the development of tomato fruit, and provides new insights into the regulation mechanisms of chlorophyll and sugar accumulation.

## Materials and methods

### Plant material and growth conditions

Tomato (*Solanum lycopersicum* L. cv. Micro Tom) plants were grown in a culture chamber under a 16/8 h light/dark photoperiod at 25 ± 2/18 ± 2 °C and 80% relative humidity.

### Sequence analysis

BLAST analysis was performed at the website http://www.ncbi.nlm.nih.gov/blast/. Protein domains were identified using the Pfam program (http://pfam.xfam.org). Alignment of protein sequences was performed with ClustalX version 2.1.

### Expression patterns and qRT-PCR analysis

Expression patterns were analysed online according to the tomato gene expression database (http://tomexpress.toulouse.inra.fr/). The pericarp of fruit was used for total RNA isolation. Total RNA was extracted using an RNeasy Plant Mini Kit (Qiagen). Quantitative real-time (qRT)-PCR was carried out as previously described by Deng *et al.* (2012). The relative transcript abundance was monitored on a CFX96 real-time PCR detection system (Bio-Rad) using All-in-One™ qPCR Mix (GeneCopoeia). The relative expression for each gene of interest was calculated using the ΔΔ*C*_t_ values with ubiquitin as an internal standard. Primer sequences are listed in Supplementary Table S1 at *JXB* online. The following genes were examined: *DDB1*, UV damaged DNA binding protein 1 (Solyc02g021650); *THY5*, bZIP domain of plant elongated/long HY5-like transcription factors and similar proteins (Solyc08g061130); *SlGLK1*, golden2-like protein 1 (Solyc07g053630); *SlGLK2*, golden2-like protein 2 (Solyc10g008160); *POR*, protochlorophyllide oxidoreductase (Solyc10g006900) *CBP1*, chlorophyll binding protein 1 (Solyc02g070990) and *CBP2*, chlorophyll binding protein 2 (Solyc02g070950).

### Subcellular localization of SlARF10

For the construction of the SlARF10-GFP fusion expression vector, the forward 5′-ATGAAGGAGGTTTTGGAGAAGTG-3′ and reverse 5′-CTATGCAAAGATGCTAAGAGGTC-3′ primers were used to amplify the sequence of *SlARF10* coded frames. Protoplasts were obtained from suspension-cultured cells of tobacco (*Nicotiana tabacum* cv. Bright Yellow-2) and transfected with the SlARF10-GFP (green fluorescent protein) fusion expression vector. Transformation assays were performed as previously described by Chaabouni *et al.* (2009).

### Generation of transgenic plants

The ORF sequence of *SlARF10* was amplified using the forward 5′-TCCCCCGGGGATGAAGGAGGTTTTGGAGAA-3′ and reverse 5′-CGGGATCCCTATGCAAAGATGCTAAGAGGTC-3′ primers. The sequence was cloned into the plant binary vector pLP100, resulting in an overexpression vector. For the construction of the RNAi vector, 200-bp sequences of *SlARF10* were amplified, and the PCR products were inserted around a spacer of the β-glucuronidase (GUS) gene in pCAMIBA2301 driven by a Cauliflower mosaic virus (CaMV) 35S promoter. Transgenic plants were generated via *Agrobacterium tumefaciens*-mediated transformation according to the methods described by Jones *et al.* (2002). All experiments were performed using homozygous lines of T3 generations.

### Chlorophyll analysis in tomatoes

Chlorophyll content was determined in the fruit pericarp and leaves according to the methods described by Powell *et al.* (2012). The experiments were performed using 2-month-old plants and the leaf samples were selected from the fourth internode counting from the shoot tip. For the determination of chlorophyll autofluorescence, the pericarp was peeled off the fruit and observed under a TCS SP2 laser confocal microscope (Leica, Germany).

### Measurement of chlorophyll fluorescence parameters

Chlorophyll fluorescence parameters were measured using a pulse-amplitude modulation fluorometer (PAM-2500, Walz, Germany). The experiments were performed using 2-month-old plants and the leaf samples were selected from the fourth internode counting from the shoot tip. The measurements were performed on leaves and green fruit, at 36 d post-anthesis (DPA), according to the methods described in detail by Maury *et al.* (1996).

### Determination of photosynthetic substances

Samples of 1 g of tomato fruit pericarp were ground in liquid nitrogen and extracted three times with 10 ml of 80% ethanol at 80 °C for 30 min. After centrifugation at 5000 *g* for 5 min, the supernatant was used for measurement of glucose, fructose, and sucrose, and the pellet was used for starch analysis. The supernatant was completely evaporated under vacuum, and dissolved in 3 ml distilled water. Sub-samples of 1 ml were used to measure glucose, fructose, and sucrose using HPLC. For starch analysis, 4 ml of 0.2 M KOH was added to the pellet and heated at 100 °C for 30 min. Each sample was added to 1.48 ml of 1 M acetic acid (pH 4.5), hydrolysed with 7 Units of amyloglucosidase for 45 min, and dissolved in 10 ml distilled water. Sub-samples of 1 ml were then used to measure starch content via HPLC.

HPLC analysis was performed using an Agilent 1260 Series liquid chromatograph system (Agilent Technologies), equipped with a vacuum degasser, a binary pump, an auto-sampler, and a diode array detector, controlled by the Agilent ChemStation software. A Waters XBridge Amide column (4.6 × 150 mm i.d., 3.5 μm) together with a pre-column (Waters XBridge BEH Amide column, 3.9 × 5 mm i.d., 3.5 μm) were employed. Separation was achieved using an isocratic solvent system that consisted of solvent A (0.2% triethylamine water solution) and solvent B (acetonitrile) as mobile phases, eluting at 75% B for 15 min. The solvent flow rate was 0.6 ml min^–1^, the column temperature was kept at 38 °C, and the injection volume for all samples was 10 μl. The detection system was an ELSD 2000 (Alltech), with a drift tube temperature of 80 °C, using air as the carrier gas with flow rate of 2.2 l min^–1^. The contents of glucose, fructose, sucrose, and starch were calculated according to the methods described by Geigenberger *et al.* (1996).

### Promoter analysis and dual-luciferase transient expression assays

The promoter sequences of *SlGLK1* and *SlGLK2* genes were analysed using the PlantCARE (http://bioinformatics.psb.ugent.be/webtools/plantcare/html/).

Transcription activity analyses of SlARF10 were conducted in leaves of tobacco (*Nicotiana benthaminana*). The full coding sequence of *SlARF10* was amplified and cloned into pGreenII 62-SK (Hellens *et al.*, 2005), which was used as an effector vector. The promoters of *SlGLK1*, *POR*, *CBP1*, and *CBP2* were cloned into the pGreenII 0800-LUC (luciferase) double-reporter vector (Hellens *et al.*, 2005). All primers used for construction of transient expression vectors are listed in Supplementary Table S1. The activities of LUC and *Renilla* (REN) luciferase were measured with a Luminoskan Ascent microplate luminometer (ThermoFisher Scientific) using a dual-luciferase assay kit (Promega) according to the manufacturer’s instructions. Six biological repeats were performed for each pair of vectors.

### Protein expression and electrophoretic mobility shift assays (EMSAs)

The B3 domain sequence (amino acid position 1–366) of SlARF10 was fused to the glutathione S-transferase (GST) tag in GEX-4T-1 (GE Healthcare Life Science) and expressed in *Escherichia coli* strain BM Rosatta (DE3). The recombinant proteins were induced with 0.8 mM isopropyl-β-D-thiogalactopyranoside for 6 h at 30 °C and purified using a GST-Tagged Protein Purification Kit (Clontech). The recombinant proteins were used for the EMSA assays with a LightShift Chemiluminescent EMSA Kit (ThermoFisher Scientific) according to the procedure described by Han *et al.* (2016). The probe containing the TGTCTC sequence from the promoter of *SlGLK1* was labelled with biotin using a Pierce Biotin 3′ End DNA Labeling Kit (ThermoFisher Scientific). An unlabeled DNA fragment of the same sequence was used as a competitor. The TGTCTC DNA fragment was modified into AAAAAA and used as a mutant probe. Biotin-labelled DNA was detected using a Chemiluminescent Nucleic Acid Detection Module Kit (ThermoFisher Scientific) according to the manufacturer’s instructions. All the primers used in the EMSA assays are listed in Supplementary Table S1.

## Results

### The *SlARF10* gene is highly expressed in tomato fruit and the SlARF10 protein is located in the nucleus

Amino-acid sequence analysis determined that SlARF10 had the domains B3-DNA, ARF, and AUX/IAA, which indicated that it had the typical conserved ARF domains (Supplementary Fig. S1).

**Fig. 1.**
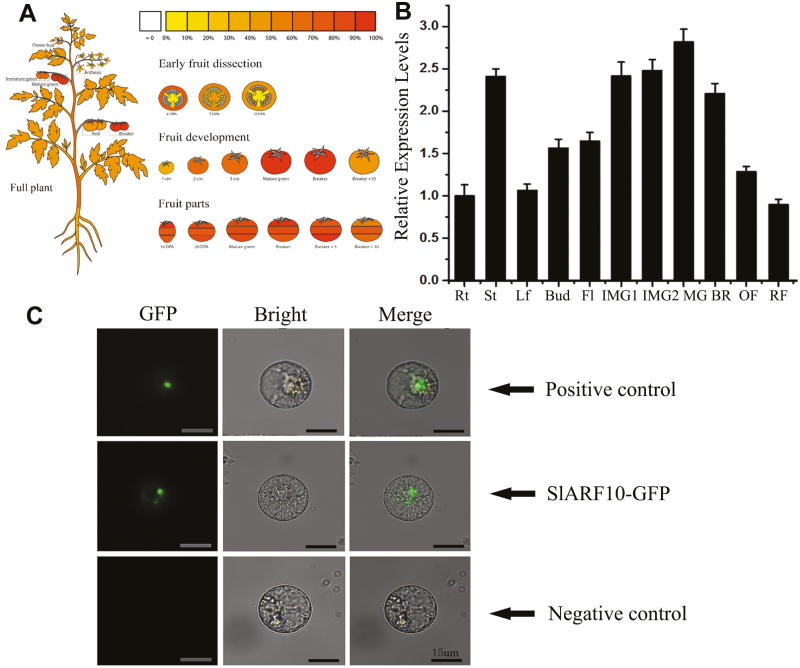
Expression patterns of the *SlARF10* gene and subcellular localization analysis of the SlARF10 protein in tomato plants. (A) Online analysis of *SlARF10* in tomato plants (see Methods). The degree of shading indicates the gene expression level. (B) qRT-PCR analysis of *SlARF10* expression levels; the housekeeping gene *ubiquitin* was used as the reference. The data are means (±SD) of three biological replicates. Rt, root; St, shoot; Lf, leaf; Fl, flower; IMG, immature green fruit (IMG1, 4 d post-anthesis, DPA; IMG2, 18 DPA); MG, mature green fruit (35 DPA); BR, breaker fruit (40 DPA); OF, orange fruit (43 DPA); RF, red fruit (46 DPA). The fruit pericarp was used for total RNA isolation. (C) Subcellular localization analysis of the SlARF10 protein. PCX-DG-GFP was the negative control. Scale bars are 15 μm.

The expression profiles of *SlARF10* in tomato were analysed via an online database and qRT-PCR. The database analysis revealed that it was expressed in all tissues, including roots, stems, leaves, flowers, and fruit. The expression level was high in fruit, especially in immature green, mature green, and breaker fruit (Fig. 1A). The qRT-PCR analysis also showed a similar expression profile (Fig. 1B). These results suggested that the *SlARF10* gene may be involved in the development of tomato fruit.

Amino-acid sequence analysis showed that SlARF10 had a nuclear-localization signal peptide. In order to verify the location in the nucleus, SlARF10-GFP fusion protein vectors were constructed and transferred into tobacco protoplasts. The green fluorescence of the fusion protein indicated that SlARF10 was located in the nucleus (Fig. 1C).

### *SlARF10* is involved in chlorophyll accumulation in tomato fruit

**Fig. 2.**
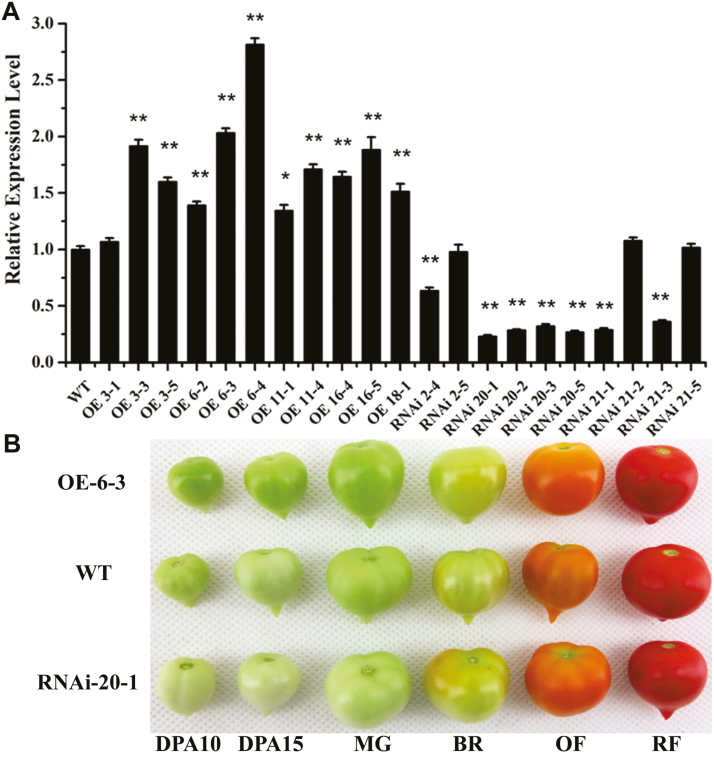
*SlARF10* transgenic plants and their fruit phenotypes. (A) qRT-PCR analysis of the expression of *SlARF10* in the transgenic lines; the housekeeping gene *ubiquitin* was used as the reference. (B) Fruit phenotypes. WT, wild-type plants; OE, SlARF10-overexpression lines; RNAi, SlARF10-RNAi lines. DPA, days post-anthesis. MG, mature green fruit; BR, breaker fruit; OF, orange fruit (2 d post-breaker stage); R, red fruit (5 d post-breaker stage). The fruit pericarp was used for total RNA isolation. Data are means (±SD) of three biological replicates. Significant differences between transgenic and WT plants were determined using Student’s *t*-test: *P<0.05, **P<0.01.

In order to examine its functions in tomato fruit development, transgenic techniques were used to up- and down-regulate *SlARF10*. Ten homozygous down-regulated transgenic lines (SlARF10-RNAi) and 11 homozygous up-regulated transgenic lines (SlARF10-OE) were generated, corresponding to independent transformation events. The T3 generation plants of the RNAi 2-4 and RNAi 20-1 lines and the OE 6-3 and OE 11-4 lines that exhibited lower and higher accumulation of *SlARF10* transcripts, respectively, were selected for further study (Fig. 2A). At the green fruit stage, the two OE lines produced fruit that were darker green than the wild-type (WT) plants whilst the two RNAi lines produced fruit that were lighter green (Fig. 2B). However, there were no visual differences in the fruit colors between the transgenic and WT plants at the subsequent breaker, orange, and red-ripe stages.

**Fig. 3.**
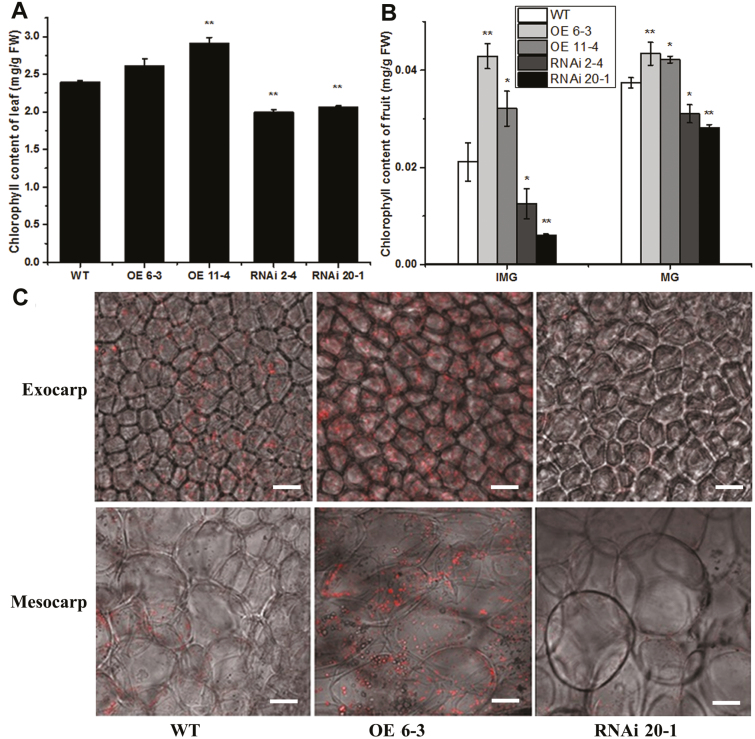
Chlorophyll accumulation in *SlARF10* transgenic plants. (A, B) Chlorophyll contents in leaves and fruit of SlARF10-overexpression (OE) and SlARF10-RNAi lines Compared with the wild-type (WT). Data are means (±SD) of three biological replicates. Significant differences between transgenic and WT plants were determined using Student’s *t*-test: *P<0.05, **P<0.01. (C) Autofluorescence of chlorophyll in the pericarp of tomato fruit, as determined by confocal laser scanning microscopy for the SlARF10-OE line 6-3 and SlARF10-RNAi line 20-1, compared with the WT. Scale bars are 10 μm.

To verify these observations, the chlorophyll contents of green fruit and leaves were determined in the plants. Compared with the WT, the RNAi lines showed lower accumulation of chlorophyll and the OE lines showed higher accumulation (Fig. 3A, B). Confocal laser-scanning microscopy was used to detect chlorophyll autofluorescence in the mesocarp (middle layer of the pericarp) and exocarp (outer layer) of the fruit. The OE 6-3 line had stronger chlorophyll autofluorescence compared with the WT plants whilst the RNAi 20-1 line had weaker autofluorescence (Fig. 3C). These results indicated that *SlARF10* is involved in chlorophyll accumulation, and that its regulation could therefore control the chlorophyll content in tomato fruit.

### Regulation of *SlARF10* expression affects the photochemical efficiency and synthesis of photosynthetic substances in tomato fruit

**Fig. 4.**
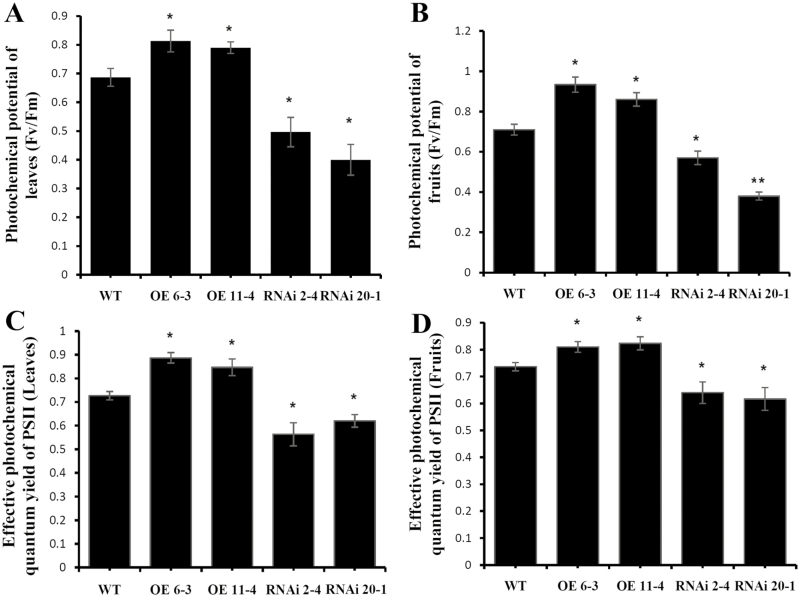
Photochemical potential in *SlARF10* transgenic plants. Photochemical potential in (A) the fruit and (B) the leaves of SlARF10-overexpression (OE) and SlARF10-RNAi plants compared with the wild-type (WT). Effective photochemical quantum yield of PSII in (C) the leaves and (D) the fruit. Data are means (±SD) of three biological replicates. Significant differences between transgenic and WT plants were determined using Student’s *t*-test: *P<0.05, **P<0.01.

Increased chlorophyll content may potentially confer higher photosynthetic performance in the transgenic plants. To assess this hypothesis, the photosynthetic performance was examined in the fruit and leaves of the selected RNAi and OE lines. Relative to the WT, the OE lines had increased photochemical potential in the fruits and leaves whilst the RNAi lines had decreased potential (Fig. 4A, B). Similar results were found for the effective photochemical quantum yield of PSII (Fig. 4C, D). Thus, regulation of *SlARF10* gene expression positively affected photosynthesis in the plants.

**Fig. 5.**
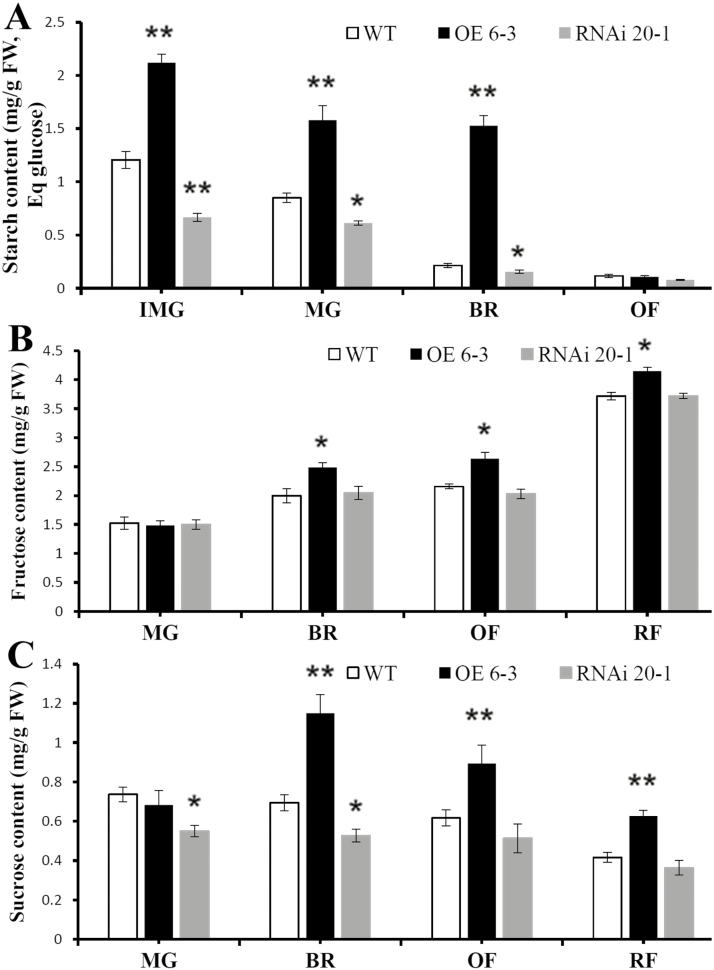
Accumulation of photosynthetic substances in the fruit of *SlARF10* transgenic plants. Contents of (A) starch, (B) fructose, and (C) sucrose in SlARF10-overexpression (OE) and SlARF10-RNAi lines compared with the wild-type (WT). Data are means (±SD) of three biological replicates. Significant differences between transgenic and WT plants were determined using Student’s *t*-test: *P<0.05, **P<0.01.

Sugars are the main products of chloroplast activity and photosynthesis, and therefore their accumulation was determined in the plants using HPLC. Starch accumulated at the immature green stage of fruit development and had decreased dramatically by the orange stage (Fig. 5A). Up-regulation of *SlARF10* increased starch accumulation in the fruit at the immature green, mature green, and breaker stages compared with the WT plants, whilst down-regulation of *SlARF10* decreased accumulation. These results indicated that regulation of *SlARF10* gene expression controlled starch synthesis during early stages of fruit development.

Starch degradation is the main source of soluble sugars and therefore we assessed the impact of *SlARF10* regulation on fructose, glucose, and sucrose in the fruit. The OE 6-3 line had significantly higher fructose contents than the WT at the breaker, orange-, and red-fruit stages, whilst the RNAi 20-1 line showed no significant differences compared with the WT plants (Fig. 5B). There were no significant differences in glucose content between the WT, OE 6-3, and RNAi 20-1 plants (data not shown). The sucrose content in the OE 6-3 line was significantly higher than in the WT at the breaker, orange-, and red-fruit stages, whilst in the RNAi 20-1 line the content was lower at the mature green and breaker stages (Fig. 5C).

### Regulation of *SlARF10* expression alters the expression of starch biosynthesis genes

**Fig. 6.**
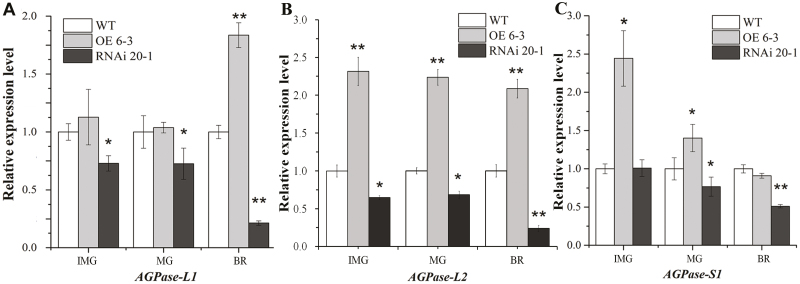
The expression of *AGPase* genes in *SlARF10* transgenic plants. The levels of transcripts were assessed in tomato fruit by qRT-PCR for (A) *AGPase-L1*, (B) *AGPase-L2*, and (C) *AGPase-S1* in SlARF10-overexpression (OE) and SlARF10-RNAi lines compared with the wild-type (WT). The housekeeping gene *ubiquitin* was used as the reference. IMG, immature green stage; MG, mature green stage; BR, breaker stage. Data are means (±SD) of three biological replicates. Significant differences between transgenic and WT plants were determined using Student’s *t*-test: *P<0.05, **P<0.01.

To gain more insight into the mechanism of sugar metabolism in the *SlARF10* transgenic plants, we examined the expression patterns of starch biosynthesis genes. AGPase genes, with four subtypes (*AGPase-L1*, *AGPase-L2*, *AGPase-L3*, and *AGPase-S1*), encode the most important enzymes in starch synthesis that catalyse the first step of the reaction. Relative to the WT, the expression of *AGPase-L1* was increased at the breaker stage in the OE 6-3 fruit and decreased in the immature green, mature green, and breaker stages of the RNAi 20-1 fruit (Fig. 6A). *AGPase-L2* showed increased expression at each of the three stages in the OE 6-3 fruit and decreased expression in the RNAi 20-1 fruit (Fig. 6B). *AGPase-S1* showed increased expression at the immature green and mature green stages in the OE 6-3 fruit and decreased expression at the mature green and breaker stages of the RNAi 20-1 fruit (Fig. 6C). The *AGPase-L3* gene showed no altered expression in the *SlARF10* transgenic lines compared with the WT plants (data not shown). Analysis of the promoter sequences in the *AGPase* genes showed that the ARF binding site, the TGTCTC box, existed in the promoters of *AGPase-L1* and *AGPase-L2* (Supplementary Table S2). The direct binding of the SlARF10 protein to the *SlGLK1* promoter was verified by EMSAs. The purified recombinant truncated SlARF10 and glutathione S-transferase (GST) fusion protein (GST-tSlARF10) were successfully obtained (Supplementary Fig. S3), but the EMSA results showed that there was no specific binding between SlARF10 and the promoters of *AGPase-L1* and *AGPase-L2*, (data not shown).

### SlARF10 positively regulates the expression of *SlGLK1*, *POR*, *CBP1*, and *CBP2*

**Fig. 7.**
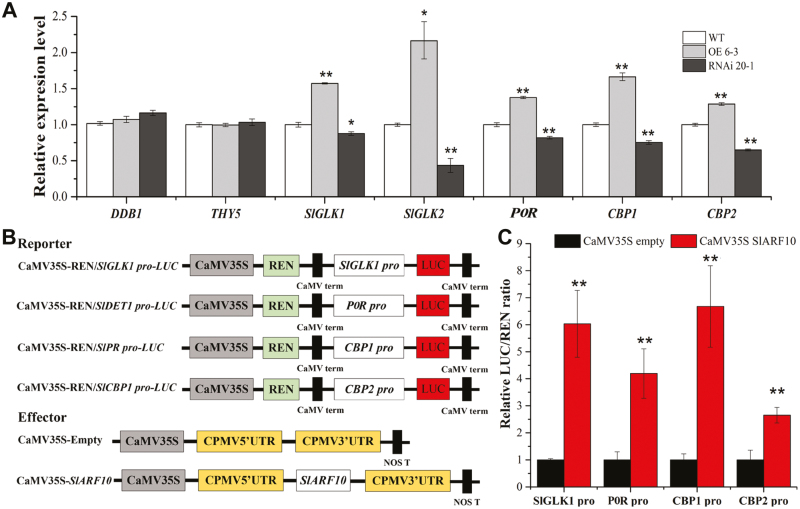
SlARF10 activates the expression of genes related to chlorophyll metabolism and regulation in tomato fruit. (A) Expression profiles of genes related to chlorophyll metabolism and regulation in SlARF10-overexpression (OE) and SlARF10-RNAi lines compared with the wild-type (WT). The housekeeping gene *ubiquitin* was used as the reference. Data are means (±SD) of three biological replicates. (B) Diagrams of the reporter and effector constructions used in the dual-luciferase reporter assay. (C) *In vivo* interactions of SlARF10 with the promoters obtained from transient assays in tobacco leaves. The ratio of LUC/REN of the empty vector plus promoter was used as a calibrator (set as 1). Data are means (±SD) of six biological replicates. Significant differences between transgenic and WT plants were determined using Student’s *t*-test: *P<0.05, **P<0.01.

The chlorophyll and starch phenotypes of the SlARF10-OE plants were reminiscent of those described in transgenic *SlGLK* overexpression plants. The expression levels of two *GLK* genes, *SlGLK1* and *SlGLK2*, were therefore examined in the OE 6-3 and RNAi 20-1 lines. qRT-PCR analysis demonstrated that there was enhanced accumulation of *SlGLK1* and *SlGLK2* transcripts in the fruit of the OE 6-3 plants and reduced accumulation of transcripts in the fruit of RNAi 20-1 plants relative to the WT (Fig. 7A). The qRT-PCR results also showed that there were no significant differences between the WT and transgenic plants in the expression levels of *DDB1* and *THY5*, which indicated that the effect of *SlARF10* on chlorophyll accumulation may have acted independently or downstream of the *DDB1* pathway. The expression levels of *POR*, *CBP1*, and *CBP2* were also examined and increased accumulation of transcripts were found in the fruit of OE 6-3 plants, whilst decreased accumulation was found in the fruit of RNAi 20-1 plants. No differences in expression of *SlARF4* were found in the OE and RNAi lines relative to the WT (Supplementary Fig. S2).

Transient expression assays were performed using the dual-luciferase reporter system to investigate whether SlARF10 could directly activate the expression of *SlGLK1*, *POR*, *CBP1*, and *CBP2*. Tobacco leaves were co-transformed with LUC reporter plasmids under the control of the promoters of *SlGLK1*, *POR*, *CBP1*, and *CBP2* together with an overexpression vector carrying *SlARF10* under the control of the CaMV35S promoter (Fig 7B). As shown in Fig. 7C, the activities of all the promoters were significantly increased in the presence of SlARF10, with relatively higher LUC/REN ratios being observed compared to the controls.

### SlARF10 targets the promoter of *SlGLK1*

**Fig. 8.**
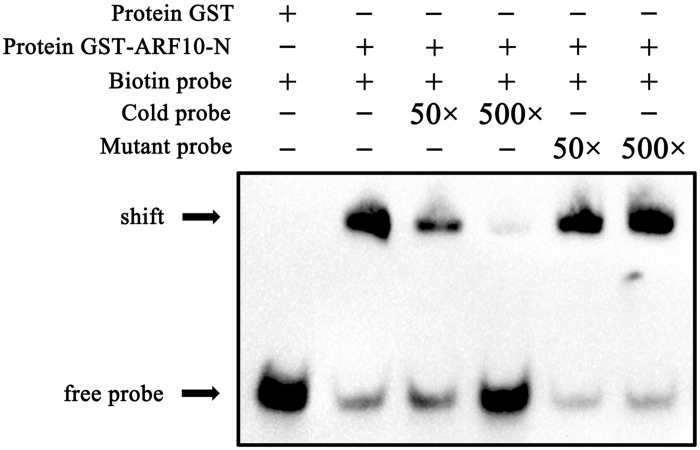
SlARF10 targets the promoters of *SlGLK1* genes. Electrophoretic mobility shift assay showing the binding of SlARF10 to the *SlGLK1* promoter. Biotin-labeled DNA probes from native promoter or mutants were incubated with the GST-SlARF10 protein, and the DNA–protein complexes were separated on 6% native polyacrylamide gels.

Analysis of the promoter sequence in the *SlGLK1* gene showed two conserved ARF binding sites, the TGTCTC box. The direct binding of the SlARF10 protein to the *SlGLK1* promoter was verified by EMSA. The GST-tSlARF10 fusion protein bound to the biotin-labeled probes containing the TGTCTC motif from the *SlGLK1* promoter and caused mobility shifts. The mobility shift was effectively abolished in a dose-dependent manner by the addition of increasing amounts of unlabeled competitors with the same sequence, but not by the mutated probes (Fig. 8). The mobility shift was also not observed when biotin-labeled probes were incubated with GST only, indicating the specific binding between SlARF10 and the *SlGLK1* promoter.

## Discussion

### SlARF10 is involved in chlorophyll accumulation in tomato fruit

Chlorophyll content, a critical feature of unripe fruit, affects the nutritional components and flavor of ripe fruit. Moreover, the link between chlorophyll content and photosynthesis (or photosynthate metabolism in fruit tissues) has been highlighted by a number of studies (Lopez-Juez and Pyke, 2005; Powell *et al.*, 2012; Nadakuduti *et al.*, 2014). However, the regulatory mechanisms by which chlorophyll impacts on photosynthetic capacity, as well as on photosynthate accumulation and thus fruit quality, remain unclear. Auxin plays a pivotal role in the initiation of fleshy fruit development and determination of final fruit size through the control and expansion of cell division (Devoghalaere *et al.*, 2012; Sagar *et al.*, 2013). Consequently, auxin impacts on an array of crucial regulators (such as ethylene, ABA, and the ripening regulator *Rin*) and vital effectors (such as genes for β-xanthophyll, lycopene biosynthesis, and chlorophyll degradation) (Su *et al.*, 2015; Manoharan *et al.*, 2017). Studies have also suggested that roots of Arabidopsis treated with auxin show increased chlorophyll accumulation and chloroplast development after detachment from shoots, and analyses of mutants indicated that auxin transported from the shoot represses chlorophyll accumulation via the function of *ARF7*, *ARF19*, and *IAA14* (Kobayashi *et al.*, 2012). A hypothesis can be drawn based on these findings in which auxin, a critical phytohormone, regulates chlorophyll accumulation and degradation via the function of ARFs during fruit setting and development.

 Given that *IAA14* and *ARF7*/*19* mediate the auxin signaling pathway to repress chlorophyll biosynthetic genes in Arabidopsis (Kobayashi *et al.*, 2012), we speculated that auxin is likely to regulate chlorophyll biosynthesis and accumulation in tomato via activated or repressed transcriptional functioning of ARFs. Previous work has shown that *DR12*/*ARF4*, a member of the tomato *ARF* gene family, influences the regulation of fruit development, such that transgenic tomato plants with down-regulated *SlARF4* expression levels bear dark-green fruit at immature stages, with a significant increase in chlorophyll content, and accumulate more starch during the colour transition stages and more sugar at the ripening stages of fruit development. *SlARF4* may function through the transcriptional repression of *GLK1* expression in tomato fruit (Jones *et al.*, 2002; Sagar *et al.*, 2013). In contrast, in our current study, up-regulation of *SlARF10*, another transcriptional repressor, resulted in enhanced chlorophyll accumulation in tomato fruit (Fig. 3). Furthermore, overexpression of *SlARF10* increased the accumulation of *SlGLK1* and *SlGLK2* transcripts in the fruit, whereas down-regulation had the opposite effect (Fig. 7). We also found that SlARF10 could positively regulate the expression of *SlGLK1* and directly target its promoter (Fig. 8). Overexpression of *SlGLK1* and *SlGLK2* is known to produce dark-green fruit (Nguyen *et al.*, 2014), which is similar what we observed in plants with *SlARF10* overexpression. Although SlGLK2 expression was increased in the SlARF10 overexpression lines and decreased in the down-regulated lines, this would not account for the dark-green phenotypes as the Micro Tom variety that we used possesses two null alleles of *SlGLK2* (Powell *et al.*, 2012). Our results indicated that *SlARF10* controls chlorophyll accumulation by regulating the expression of *SlGLK1*, with SlARF10 functioning as a transcriptional activator of *SlGLK1*. *SlGLK1* and *SlGLK2* have similar functions, but different expression patterns effectively restrict *SlGLK1* mostly to leaf functions and *SlGLK2* mostly to fruit functions in tomato plants (Nguyen *et al.*, 2014). Our results indicated that *SlGLK1* may have important functions in chlorophyll accumulation not only in tomato leaves, but also in the fruit, and in future studies knock-out of *SlGLK1* in the fruit could be performed to further elucidate its roles in chlorophyll accumulation in tomato plants. It was interesting that the expression levels of *SlARF4* were not changed in the SlARF10-OE and SlARF10-RNAi lines (Supplementary Fig. S2). Thus, the data suggest that regulation of *SlGLK1* expression by *SlARF10* may act independently of SlARF4.

Our results showed no significant differences between the WT and transgenic plants in the expression levels of *DDB1* and *THY5* (Fig. 7). In the dark, active DDB1 causes the degradation of photomorphogenesis-promoting transcription factors such as THY5. Exposure to light represses *DDB1* and activates *THY5*, leading to transcriptional activation of photomorphogenesis and pigment genes (Cocaliadis *et al.*, 2014). The regulation of chlorophyll accumulation by *SlARF10* may act independently or downstream of the *DDB1* pathway. Chlorophyll *a* is initially synthesized from glutamyl-tRNAglu, and chlorophyll *b* is synthesized at the final step of chlorophyll biosynthesis. Analysis of the complete genome of Arabidopsis has determined that there are 15 enzymes encoded by 27 genes for chlorophyll biosynthesis (Beale, 1999). Our study showed that SlARF10 positively regulated the expression of *POR* (Fig. 7), which encodes a protochlorophyllide oxidoreductase that catalyses protochlorophyllide into chlorophyllide, a key step in chlorophyll biosynthesis (Cahoon and Timko, 2000). Thus, SlARF10 may directly regulate the expression of this key gene, thereby influencing chlorophyll biosynthesis at the developmental stages of tomato fruit. We also found that SlARF10 positively regulated the expression of *CBP1* and *CBP2*. Chloroplast membranes contain many kinds of chlorophyll binding proteins and they are associated with chlorophyll and xanthophylls, which absorb sunlight and transfer the excitation energy to the PSII core complexes to drive photosynthetic electron transport (Hiller *et al.*, 1995). The increased expression of *CBP1* and *CBP2* may explain the improved photochemical efficiency in the *SlARF10* overexpression lines (Fig. 4).

### Altering *SlARF10* expression affects accumulation of photosynthates in tomato fruit

*SlARF10* up-regulated lines display dark-green fruit phenotypes similar to those seen in *SlARF4* down-regulated lines with enhanced chlorophyll content (Sagar *et al.*, 2013). In contrast to *SlARF4* down-regulated plants where the dark-green phenotype is restricted to immature fruit, significantly higher chlorophyll content was detected in leaf and fruit tissues of the *SlARF10*-OE lines (Fig. 3). This indicated that, in contrast to *SlARF4*, *SlARF10* control of chlorophyll accumulation was not fruit-specific. Furthermore, the higher chlorophyll content in the *SlARF10*-OE lines correlated with higher photochemical efficiency compared with the WT plants (Fig. 4), and resulted in elevated starch and sugar contents in the transgenic fruit (Fig. 5). Starch is not only a significant carbohydrate reserve in the majority of plants but also a major factor in defining fruit nutrition and flavor. In starch synthesis, the first regulatory step (the synthesis of ADP-glucose) is catalysed by AGPase from glucose-1-phosphate and ATP (Stark *et al.*, 1992; Yin *et al.*, 2010). In potato (*Solanum tuberosum*) tubers, it has been shown that this critical catalytic reaction is also the limiting step during starch biosynthesis (Tiessen *et al.*, 2002). Auxin regulates expression of *SlAGPase* genes (Miyazawa *et al.*, 1999). Down-regulation of *SlARF4* increases both starch content and the expression of essential genes involved in starch biosynthesis in tomato fruit, particularly genes coding for AGPase (Sagar *et al.*, 2013). In our study, the improved starch content in the *SlARF10*-OE lines correlated well with increased expression of *AGPase* genes in starch biosynthesis (Fig. 6). The promoter regions of *AGPase S1* and *AGPase S2* have auxin-responsive motifs (Supplementary Table S2); however, the EMSA data showed that SlARF10 did not directly bind to the promoters of these genes, indicating that *SlARF10* regulates starch accumulation by indirectly controlling the expression of *SlAGPase* genes.

Many studies of tomato have found that the vast majority of photoassimilates in the fruit are supplied by the leaves rather than produced *de novo* in the fruit (Do *et al.*, 2010; Ruan *et al.*, 2012): more than 80% of the total carbon of the fruit has been estimated to result from photosynthetic activity in the leaves (Cocaliadis *et al.*, 2014). In this study, it is possible that the enhanced leaf photosynthesis observed in the *SlARF10*-OE lines improved the transportation of photoassimilates into the fruit, resulting in the increased starch content. Wang *et al.* (2005) reported that down-regulation of *SlIAA9* alters auxin sensitivity and facilitates the development of vascular bundles, thereby probably increasing sink strength as well as the supply of assimilates to the fruit. Up-regulation of *SlARF10* also resulted in higher fructose content at various stages of fruit development (Fig. 5), probably due to the increased starch content, which was available to be degraded into soluble sugars This finding was in accordance with previous studies demonstrating that starch content in the early stages of development determines the soluble solid content in mature fruit (Schaffer *et al.*, 2000; Baxter *et al.*, 2005). In our study, up-regulation of *SlARF10* produced higher sucrose content, whereas down-regulation lines displayed decreased sucrose accumulation at some stages of tomato fruit development (Fig. 5). Sucrose is produced in photosynthetically active tissues (sources) and translocated to non-photosynthetic tissues (sinks) (Bihmidine *et al.*, 2013). Li *et al.* (2002) reported that sucrose significantly elevates the expression of *AGPase* genes in tomato leaves and fruit. The increased starch accumulation in the *SlARF10*-OE lines may also be explained by the sucrose-enhanced expression of *AGPase* genes in the fruit.

It has been reported that miR160 targets and represses the *SlARF10* gene; however, it has very low expression in fruit at the mature green, breaker, and red stages, and in leaves (Hendelman *et al.*, 2012). In our study, SlARF10 driven by the 35S promoter in mature green fruit had higher expression levels compared with the WT plants (Fig. 7). The effects of *SlARF10* overexpression on the accumulation of chlorophyll and sugars in the fruit and leaves may also demonstrate the low activity of miR160 during their development.

In summary, our study demonstrates that *SlARF10* plays a significant role in chlorophyll accumulation during fruit development in tomato plants. Our results also shed some light on the ability of auxin to regulate starch accumulation during fruit development by altering the expression of *SlARF10*. However, auxin regulation of carbohydrate accumulation, especially its connection with other regulatory mechanism, remains to be determined. Future studies could focus on examining the auxin regulatory network for chlorophyll and starch biosynthesis, and determining the gene functions of relevant transcriptional factors.

## Supplementary data

Supplementary data are available at *JXB* online.

Fig. 1. Sequence comparison of SlARF10 and AtARF10.

Fig. 2. Relative expression levels of *SlARF4* in *SlARF10* transgenic lines.

Fig. 3. SDS-PAGE gel demonstrating affinity purification of the recombinant GST-SlARF10 protein used for EMSAs.

Table S1. Primers used for qRT-PCR, vector construction and EMSA.

Table S2. ARF binding element (TGTCTC) in AGPase genes.

Supplementary Tables

Supplementary Figures

## Abbreviations:

ARF: auxin response factor
B3: B3 DNA-binding domain
CBP1: chlorophyll binding protein 1
CBP2: chlorophyll binding protein 2
DDB1: UV-DAMAGED DNA-BINDING PROTEIN 1
GLK: GOLDEN2-LIKE
qRT-PCR: quantitative real-time PCR
WT: wild-type
