# Supplementary material for: SlARF10, an auxin response factor, is involved in chlorophyll and sugar accumulation during tomato fruit development
Source: J Exp Bot. 2018 Sep 13;69(22):5507–18. doi: 10.1093/jxb/ery328 (PMC6255703; doi:10.1093/jxb/ery328)
Supplement: Supplementary Figures [file ery328_suppl_supplementary_figures.pdf]

SlARF10, an auxin response factor, is required for chlorophyll and sugar accumulation during tomato fruit development. *Yujin Yuan, Lihua Mei, Mengbo Wu, Wei Wei, Wei Shan, Zehao Gong, Qian Zhang, Fengqing Yang, Fang Yan, Qiang Zhang, Yingqing Luo, Xin Xu, Wenfa Zhang, Mingjun Miao, Wangjin Lu, Zhengguo Li, and Wei Deng*

SUPPLEMENTARY DATA

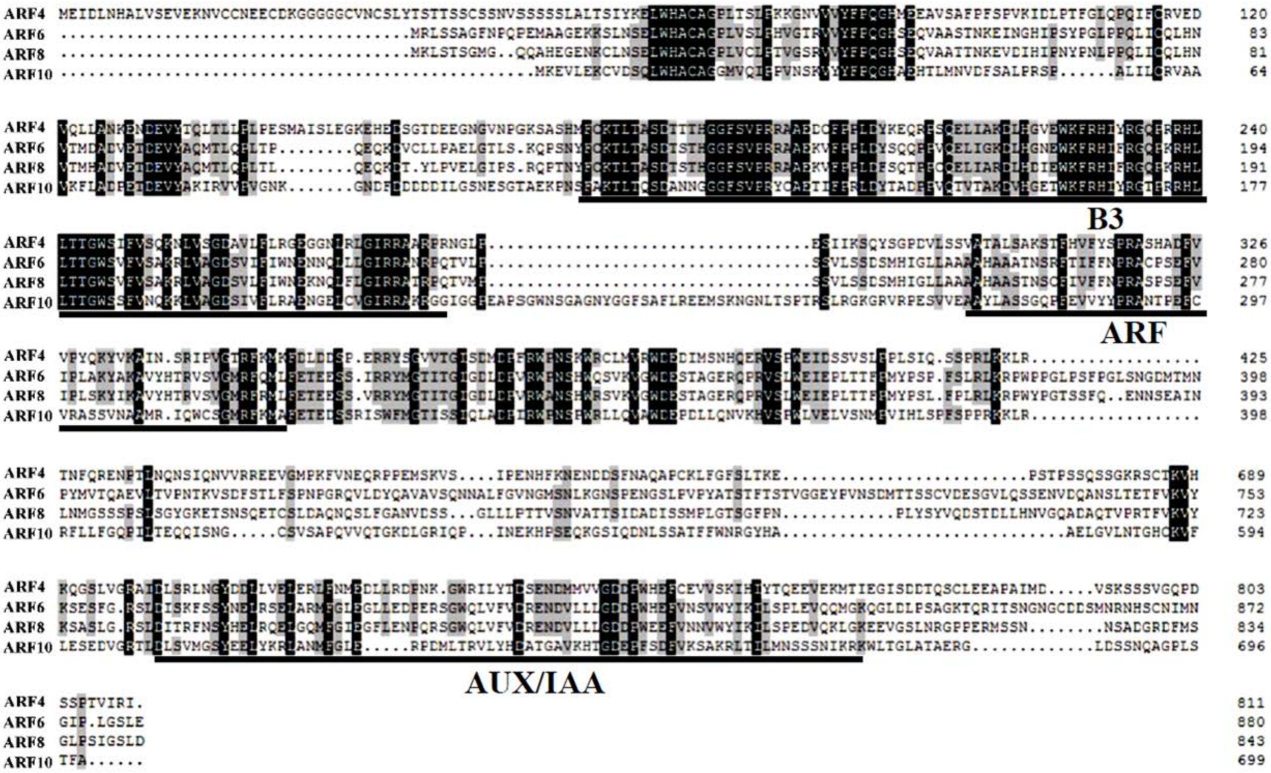

Supplementary Fig. 1. Sequence comparison of SlARFs of tomato plants.

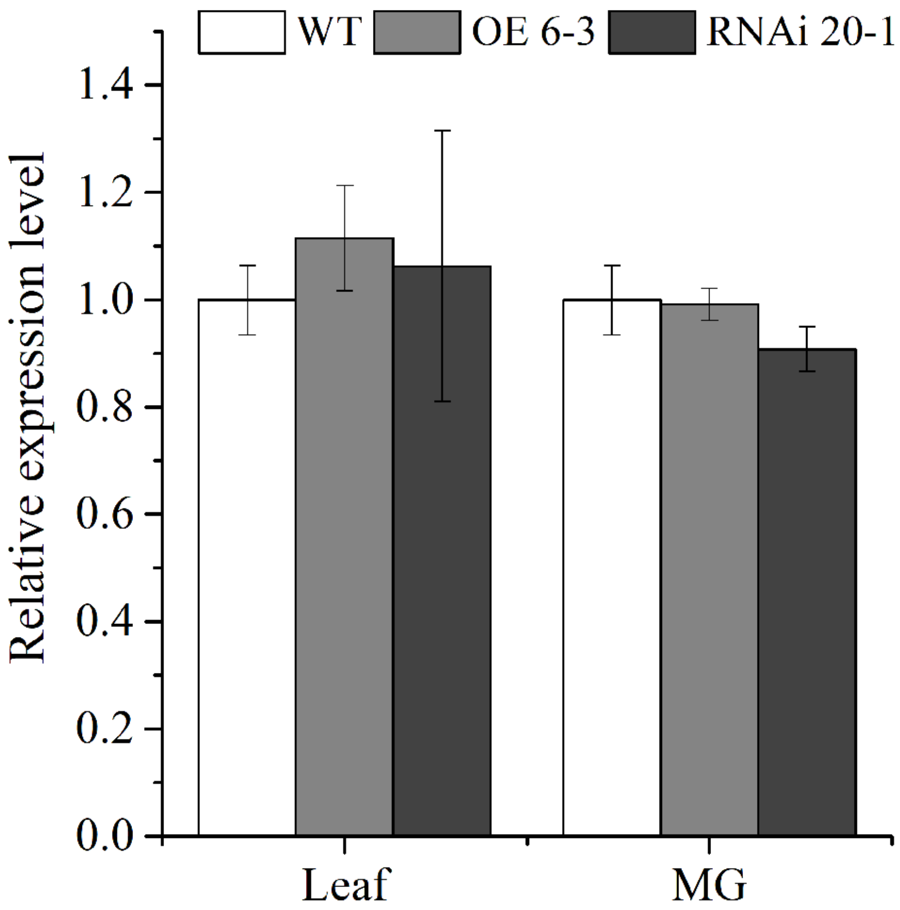

Supplementary Fig. 2. Relative expression levels of SlARF4 in SlARF10 transgenic lines.

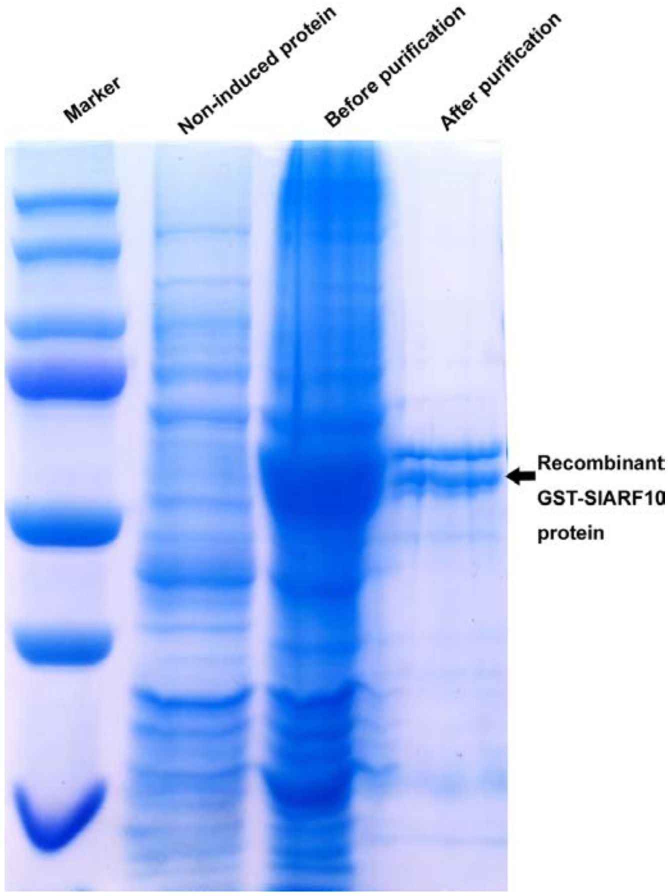

Supplementary Fig. 3. SDS-PAGE gel stained with coomassie brilliant blue demonstrating affinity purification of the recombinant GST-SlARF10 protein used for the EMSA assay.
